# Chagas Disease: Seroprevalence and Associated Factors in Indigenous Communities of the Southern Limit of Argentine Chaco

**DOI:** 10.3390/tropicalmed8010064

**Published:** 2023-01-14

**Authors:** Carlina Colussi, Marcelo Nepote, Romina Chiaraviglio, Diego Mendicino

**Affiliations:** 1 Centro de Investigaciones sobre Endemias Nacionales, Facultad de Bioquímica y Ciencias Biológicas, Universidad Nacional del Litoral, Santa Fe S3001XAI, Argentina; 2 Programa Provincial de Control de Chagas, Ministerio de Salud de Santa Fe, Santa Fe S3000GVX, Argentina; 3 Consejo Nacional de Investigaciones Científicas y Técnicas, Santa Fe S3001XAI, Argentina

**Keywords:** chagas disease, Chaco, indigenous communities, seroprevalence, risk factors

## Abstract

Chagas disease is more prevalent in socially vulnerable communities in the Gran Chaco Eco-region. The study evaluated the seroprevalence of Chagas disease and associated factors between May 2014 and September 2015, in indigenous communities of Santa Fe, Argentina, in the southern Chaco. Lysate ELISA and indirect hemagglutination tests were used to detect antibodies against *Trypanosoma cruzi*, and recombinant ELISA was used in the case of disagreement. Household surveys were conducted with the head of household about risk factors for the disease. Serological tests were conducted on 298 people from three indigenous communities, 127 male and 171 female. Seroprevalence was 18.5%. A total of 64 surveys were conducted; 82.8% of the heads of household were male, with a median age of 39 years, and 61.0% had not completed primary school. In 35.9% of the households, there was at least one member of the cohabiting group infected with *T. cruzi*. The level of education of the head of household showed a statistically significant association with Chagas disease (OR = 3.43), among all the risk factors studied. The prevalence of infection is lower than that of other indigenous communities of the Gran Chaco, probably because environmental conditions are moderating and disfavoring the establishment of the insect vector in homes, but also because of socioeconomic differences with the rest of the eco-region. Beyond this, serological controls are needed to prevent vertical transmission.

## 1. Introduction

Chagas disease, caused by the protozoan *Trypanosoma cruzi*, continues to be one of the main public health problems in Latin America. It is estimated that approximately one-quarter of those infected worldwide live in Argentina [1]. The eco-region of the Gran Chaco (North of Argentina, East of Bolivia, West of Paraguay, and a small part of Brazil) is historically the most prevalent, due to the presence of the insect vector *Triatoma infestans* (known as *vinchuca* in Argentina), adapted to live in precarious rural housing and coexisting with human populations. In addition to environmental characteristics, social, economic, and cultural factors favor the risk of infection and the prevalence of the disease. As we move away from this eco-region, risk and prevalence decrease. Indigenous communities in the region are the most vulnerable, due to their current and historical living conditions [2].

Beyond Latin America, Chagas disease has spread to countries where the insect vector is not present, due the migration of infected people, and it has been increasingly detected in the United States of America, Canada, and many European and some African, Eastern Mediterranean, and Western Pacific countries. Furthermore, in Latin America, the distribution of Chagas disease was modified due to the migration of people from a rural to urban setting, although the vector transmission was controlled [1].

The vertical transmission route (from an infected mother to her children) is of great importance and, in addition, is the one that generates the largest number of new cases in areas where the vector route is controlled [3]. For both the vector and the vertical routes, family dwellings constitute clusters of infected individuals, either because they live with the same risk due to the presence of the insect in their homes, or because mothers can transmit it to their children, having even demonstrated cases of second-generation infection [4,5]. The oral route of transmission, which could also be important at family clusters, has not been reported in Argentina.

A randomly selected study of 18 year old men in Santa Fe Province, Argentina, between 1981 and 1994 found that the prevalence of the disease decreased from the north (Chaco region of Santa Fe) to the south (Espinal and Santa Fe Grassland regions). When the risk factors that influenced this difference in prevalence were investigated, it was found that the distribution coincided with social and economic factors, with the Chaco region being the most disadvantaged [6]. A more recent seroprevalence study found 18.32% of Chagas disease in indigenous communities of the southern Gran Chaco, but risk factors were not evaluated [7].

On the basis of this epidemiological background and the lack of current studies on the association between the seroprevalence and risk of Chagas disease in inhabitants and cohabiting groups, belonging to indigenous communities of the South of the Chaco, the objective of the study was to verify these associations in the Santafesino Chaco.

## 2. Materials and Methods

### 2.1. Study Area

The study was conducted in indigenous communities in the south of the Chaco, in the province of Santa Fe (Argentina). The south of the Chaco is characterized by a warm subtropical climate, with average annual temperatures that decrease from north to south, from 23 °C on the border with Paraguay to 18 °C in the center of the province of Santa Fe. Rainfall decreases from east to west between 1300 and 750 mm per year. The predominant vegetation type is xerophilous deciduous forest [8].

**Figure 1:**
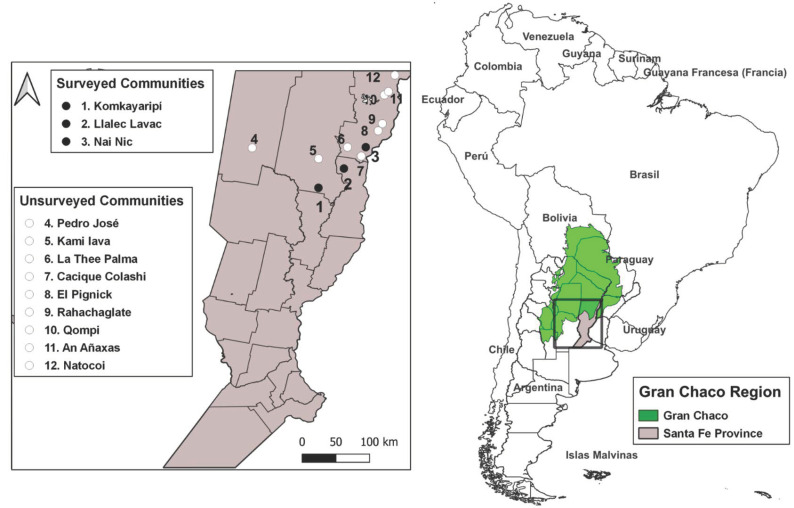
Map of geographic localization of the southern Chaco surveyed communities.

In this region, there are 13 communities registered in the Provincial Santafesino Institute of Aborigines [9], 10 of the Moqoit ethnic group and three of the Qom ethnic group. Through previous contacts, three of the Moqoit communities were included in the present work: Komkairipí (29°53′34″ S 60°16′00″ W), Lallec Lavac (29°33′29″ S 59°55′37″ W), and Nai Nic (29°08′40″ S 59°38′38″ W) (Figure 1). Due to the difficulties in physical access by road, in other communities, only serological analyses could be carried out, but they could not be included in the present work.

### 2.2. Data Collection

The leaders of the three communities were previously contacted to inform them about the objectives, the methodology of the study, and its importance. Subsequently, meetings were held during which this information was transmitted to the communities.

The serological and risk factor survey was conducted during home visits, conducted between May 2014 and September 2015. In the survey, one person over 18 years of age per household was surveyed, and serological analyses for Chagas was offered to anyone over 1 year of age who voluntarily accessed them. Informed consent was requested for blood sampling and serological analysis. In the case of minors under 18 years of age, the consent of their legal representative was requested.

### 2.3. Serological Analysis

Approximately 5 mL of blood was drawn by venipuncture with sterile needles and syringes. The sample was collected in sterile tubes with coagulation activator and separator gel (BD Vacutainer); the clot was allowed to form for 15 min at room temperature and centrifuged in the field at 3500 revolutions per minute (rpm) for 10 min. The disposable material was transported to the laboratory for disposal, following current biosafety standards.

Samples were kept in the primary tubes refrigerated at 4–8 °C during transfer to the laboratory, stored at 4–8 °C, and processed within 7 days. After processing, aliquots were separated from each sample and stored at −20 °C.

All samples were analyzed by indirect hemagglutination (Chagatest HAI, Wiener Lab, Rosario, Argentina) and lysate ELISA (Chagatest Lysate ELISA, Wiener Lab, Rosario, Argentina), following the manufacturer’s instructions. In case of disagreement, recombinant ELISA (Chagatest recombinant ELISA v.3.0, Wiener Lab, Rosario, Argentina) was performed. For the processing of the ELISA tests, a Micropar Washer (Rosario, Argentina) and Mindray MR-96A reader (Shenzhen, China) were used. A positive result was considered when two of the tests were positive, according to recommendations from the Pan American Health Organization [10].

Serological analyses were carried out in the laboratory of the National Endemic Research Center of the National University of the Litoral, which has external quality controls of the National Institute of Parasitology “Dr. Mario Fatala Chabén”.

The results were reported in writing individually to each participant, and in compiled and anonymous form to the corresponding Health Center and the Provincial Chagas Control Program.

### 2.4. Risk Factors Survey

The survey on risk factors consisted of 23 questions, in a nonlogical sequence to avoid inducing the answers of the study participants. The questions were elaborated on the basis of previous work [11,12,13]. The approximate duration was 30 min per survey. Five questions were aimed at characterizing the head of household, 13 were aimed at assessing the risk present in the dwelling, and five were aimed at determining the knowledge of the head of household about the disease.

To inquire if the vector insect was recognized, photographs were shown with different insects from the area. In relation to the disease, knowledge about the transmission routes was considered sufficient if the vectorial and vertical routes were mentioned, knowledge about the clinical aspects was considered sufficient if the possibility of cardiac involvement was indicated, and knowledge about the treatment was considered sufficient if the possibility of treatment was indicated.

In relation to risks, the presence of animals was considered to include both pets and farm animals, at home and peri-domiciliary. A house was considered risky when somewhere in its construction it had walls of mud, wood, and/or cane and/or a roof consisting of branches and/or straw [14]. Critical overcrowding was considered when three or more people lived per room.

For the analysis of the associations between households with at least one infected with *T. cruzi* and the maximum level of education reached by the head of household, the sample was divided into two groups: up to incomplete primary school and at least complete primary school.

### 2.5. Statistical Analysis

Serology and risk factor survey data were entered into an Excel spreadsheet and analyzed using InfoStat^®^ (Cordoba, Argentina) [15] and EpiInfo 7.2.1. ^®^ (Atlanta, EEUU) [16].

Every quantitative variable was analyzed by its distribution, using t-Student Test (parametric) and U Mann Whitney Test (non parametric). As the population age variable presented a nonparametric distribution, it was described as the median and range. In addition, categorical variables, such as the prevalence of *T. cruzi* infection in women of childbearing age, from 15 to 44 years, were presented as a frequency distribution.

The measures of association were calculated using a 2 × 2 contingency table, the adjusted chi-square test, the odds ratio (OR), and the corresponding confidence intervals (CI). Statistical significance was considered at a *p* ≤ 0.05.

## 3. Results

### 3.1. Study Population

A total of 298 people from the 863 belonging to the three communities participated in the serological study, corresponding to 34.5% of the population: 38.5% from Komkayaripí (185/480), 22.2% from Llalec Lavac (54/243), and 42.1% from Nai Nic (59/140). A seroprevalence for Chagas disease of 18.5% (55/298) was found (Table 1).

### 3.2. Seroprevalence of T. cruzi Infection

Overall, 57.4% of participants were female (171/298) and 42.6% were male (127/298). Furthermore, 17.5% of females (30/171) and 19.7% of males (25/127) were seropositive. No statistically significant difference was found for infection by sex (OR: 1.15; CI: 0.64–2.07; χ^2^: 0.22; *p* = 0.64). Of the 171 women tested serologically, 76 were of childbearing age. Seroprevalence in women of childbearing age was 31.6% (24/76).

Of the total number of seropositive patients, only five had been previously examined for Chagas disease and did not report symptoms or receive etiological treatment. None of the patients had a story of having migrated from another region; however, they moved within the Santafesino Chaco. All seropositive patients were referred to the corresponding primary healthcare center for clinical evaluation and/or treatment according to the guidelines of the Pan American Health Organization [10].

### 3.3. Risk Factors Related to T. cruzi Infection

A total of 64 surveys were conducted in 39.3% (64/163) of households. The median number of inhabitants per dwelling was 3.5. In 35.9% (23/64) of the dwellings, there was at least one seropositive inhabitant.

Regarding the sociodemographic data of the heads of household, age, sex, and level of schooling were evaluated. The results can be seen in Table 2.

In relation to knowledge about the vector and the disease, 90.6% (58/64) of respondents recognized the kissing bug as the transmitting insect, 31.3% (20/64) of respondents identified the transmission routes, 65.6% (42/64) recognized the cardiac form as probable, and 48.4% (31/64) knew that there was etiological treatment.

### 3.4. Association Analysis

The associations between housing with at least one infected with *T. cruzi* (positive housing) and schooling of the head of household, presence of animals in the dwelling, critical overcrowding, characteristic of the dwellings, storage of firewood, and observation of triatomines in the dwelling are summarized in Table 3. The maximum schooling achieved by the head of household was divided into two groups: up to incomplete primary and complete primary school or more.

When analyzing the association between the independent variables and positive housing, a statistically significant association was only found with the maximum schooling of the head of household (*p* = 0.03).

## 4. Discussion

This paper highlights the epidemiological situation of Chagas disease in indigenous communities of the southern Chaco. Having studied 34.5% of the population and 39.3% of the dwellings of three of the 11 communities, *T. cruzi* infection was found in approximately one in five inhabitants, and at least one infected inhabitant was found in one in three dwellings.

The situation of the three communities in which work was carried out may not be representative of the totality of the indigenous communities in the region. On the one hand, there are also minority communities of Qom ethnicity in the region, which do not necessarily share the social and cultural characteristics that imply risk for the transmission of Chagas disease. On the other hand, this activity was developed with these three communities due to the feasibility of access, and it should not be ruled out that, in the most remote communities and with greater difficulties to access, the risks and seroprevalence are different. In this sense, a seroprevalence study in the region [7] found that Chagas disease was more frequent in urban communities than in rural ones, because people with the worst socioenvironmental conditions migrated more frequently to urban settings and, therefore, these people were the most at risk of infection. This point may also explain the higher seroprevalence found in the present work compared to [7], in which urban and rural communities were studied. In the present work, only the communities with greater ease of access, which are urban or close to urban centers, were included, and these communities would have a higher seroprevalence.

The prevalence of infection found in the present study was lower than that of others carried out in indigenous communities of the Gran Chaco [17,18,19], probably because the environmental conditions are improving and becoming less favorable for the establishment of the vector insect in the homes, in a transition from a hot, semiarid lowland region to a humid subtropical climate in the spinal and grassland region. Socioeconomic differences with the rest of the eco-region could also drive this lower prevalence, mainly with the reduction in poverty [20]. These authors also found that the prevalence in indigenous communities was higher than the prevalence in Creole communities, for social and cultural reasons, despite sharing the same environmental conditions [17,18,19].

When analyzing housing factors, such as the material of its construction, overcrowding, and the presence of firewood or animals that are considered a risk for the presence of infection [21], this association was not corroborated in this study. The Santa Fe province was certified by the Pan American Health Organization as free of vector-borne transmission in 2012; for this purpose, periodic home fumigation campaigns were carried out [22], among other measures. Although, in the present work, it was found that the risk of infestation is present, the residual effect of insecticides would keep homes free of infestation for the time being; hence, the prevalence found is due to the fact that the infection is chronic, and serology remains positive for life. In this sense, other authors [7] found greater seroprevalence in urban populations than in rural ones, due to the migration to the cities of families that had worse social and sanitary conditions in rural areas.

In most homes, a man aged 39 or older was recognized as the head of household due to ethnic cultural patterns. In addition, in most cases, the head of household had not completed primary schooling, which conditioned their job possibilities, as well as the income level of the family group, and which would increase the risk of contracting the infection [23]. The maximum level of education attained by the head of household was the only risk factor studied that showed a statistically significant association with the presence of infection. There was a 3.43-fold greater likelihood of finding an infected family member in households whose head of household had not completed primary school. Similar results were found in the Qom and Creole communities of Chaco, where, with fewer than 6 years of schooling, the rate of home infestation was 30.1%, whereas, when it increased to more than 6 years of schooling, the infestation rate decreased to 19.0%. On the other hand, other authors [24] found that, in people infected with *T. cruzi* with a lower level of education, the risk of progression of heart disease increased, whereby a lower level of schooling was correlated with a greater risk of becoming infected and a worse prognosis of the disease. The level of education has a positive impact on healthcare, on changing behaviors, and on the access to and effective and timely use of health services, constituting a social determinant of health that affects the quality of life of individuals, their level of health, and therefore, human development.

Although the province of Santa Fe was certified as free of vector transmission, the prevalence found indicates the importance of seroprevalence studies. Pan American Health Organization estimated that 70% of the people living with Chagas disease do not know they are infected [25]. Similar results were found in this study, in which only five patients knew they were infected. In women of childbearing age, it is important to screen for Chagas disease and treat seropositive women, as it has been shown to reduce the chances of transmission to their offspring [26]. In the same vein, tests should be carried out on children born to seropositive mothers in order to detect and treat those infected early [27]. Taking into account the change in the epidemiological distribution of Chagas disease, with the increasing spread to urban areas and outside the continental area of Latin America, screening for this infection is also important outside the area historically considered at risk for this disease, to clinically assess those affected and treat them if recommended. The results of this study show that the majority of the population recognizes the insect vectors; however, vertical transmission and treatment are less known. Therefore, innovative strategies are necessary to train the community on this disease, adapted to their level of education and respecting their cultural patterns [28]. The main points on which prevention must work are the need for serological controls in pregnant women and children of seropositive mothers, and the importance and availability of specific treatment.

## 5. Conclusions

In the indigenous communities of the south of the Chaco, the prevalence of Chagas is lower than in the Gran Chaco and the vector situation can be considered under control. Campaigns for the detection and treatment of infected people must be continuous over time, and training activities and improvements in living conditions, as well as general education, in these communities must be the main points of health policies with community participation, not only to reduce the risks of infection, but also to promote participation and empowerment in the health decisions of these communities.

## Figures and Tables

**Table 1:** Frequency of seropositivity for Chagas by communities and sex. Indigenous communities of the southern Chaco 2014–2015.

Community	%
Komkayaripí	21.1 (39/185)
Llalec Lavac	11.1 (6/54)
Nai Nic	17.0 (10/59)
Sex	
Female	17.5 (30/171)
Male	19.7 (25/127)
Total	18.5 (55/298)

**Table 2:** Age, sex, and level of schooling of the head of household by community. Indigenous communities of the southern Chaco (2014–2015).

Communities	Age (Years)		Sex		Schooling				
	Median	Rank	Female	Male	I	IP	CP	IS	CS
Komkayaripí	44.5	23-73	15.0% (6/40)	85.0% (34/40)	17.5% (7/40)	45.0% (18/40)	25.0% (10/40)	7.5% (3/40)	5.0% (2/40)
LlalecLavac	35.0	25-59	11.1% (1/9)	88.9% (8/9)	11.1% (1/9)	33.3% (3/9)	55.6% (5/9)	0% (0/9)	0% (0/9)
Nai Nic	38.0	28-73	26.7% (4/15)	73.3% (11/15)	20.0% (3/15)	46.7% (7/15)	6.7% (1/15)	13.3% (2/15)	13.3% (2/15)
Total	39.0	23-73	17.2% (11/64)	82.8% (53/64)	17.2% (11/64)	43.8% (28/64)	25.0% (16/64)	7.8% (5/64)	6.3% (4/64)

I: illiterate, IP: incomplete primary, CP: complete primary, IS: incomplete secondary, CS: complete secondary.

**Table 3:** Frequency of the head of household’s level of schooling and housing variables, and their association with positive housing. Indigenous communities of the southern Chaco (2014–2015).

Variable	Positive Housing		Negative Housing		Frequency Total (%)	Odds Ratio	CI 95%	*p*-Value
	N	%	N	%				
Schooling of the head of household								
Incomplete primary	18	46.2	21	53.8	43.8	3.43	1.11–10.59	0.03
Complete primary	5	20.0	20	80.0	25.0			
Presence of animals								
Yes	21	35.6	38	64.4	87.5	0.83	0.15–4.57	0.84
No	2	40.0	3	60.0	12.5			
Critical overcrowding								
Yes	18	40.9	26	59.1	68.8	2.08	0.66–6.49	0.22
No	5	25.0	15	75.0	31.3			
Characteristic of the house								
With risk	5	38.5	8	61.5	20.3	1.15	0.34–3.86	0.83
Without risk	18	35.3	33	64.7	79.7			
Firewood storage								
Yes	14	46.7	16	53.3	46.9	2.43	0.87–6.78	0.09
No	9	26.5	25	73.5	53.1			
Observation of kissing bugs								
Yes	7	31.8	15	68.2	12.5	0.76	0.26–2.20	0.61
No	16	38.1	26	61.9	87.5			

## Data Availability

The data presented in this study are available on request from the corresponding author.
